# Procedure and Treatment Refusal in Pediatric Practice: A Single-Center Experience at a Children’s Hospital in Saudi Arabia

**DOI:** 10.7759/cureus.80936

**Published:** 2025-03-21

**Authors:** Ebtehal S Alharbi, Assia S Alwabel, Nada K Algaith, Razan S Alqarzaee, Rawan H Alharbi, Samah F Alkuraydis, Ibrahim A Alrashidi

**Affiliations:** 1 Pediatric, College of Medicine, Qassim University, Qassim, SAU

**Keywords:** intervention, procedure, refusal, refusal form, treatment refusal

## Abstract

Introduction: Refusal of procedures and treatment for ill children aged up to 14 years old remains a significant issue in the pediatric service. When families refuse medical treatment for their ill children for various reasons, such as financial or transportation difficulties, and concerns about severe side effects, healthcare professionals may attempt to provide information and negotiate with the family about treatment and procedure refusal and its potential consequences, as well as request consent for treatment refusal. In Saudi Arabia, there is a need for more data on treatment refusal.

Objective: We aim to determine the frequency of refusal of medical procedures and treatment in pediatric patients at a children's hospital in Qassim, Saudi Arabia.

Methods: A retrospective review of 1,296 medical records were included. All patients were less than 14 years old. Data were extracted from the hospital's database system. Data analysis was carried out using Statistical Product and Service Solutions (SPSS, version 24; IBM SPSS Statistics for Windows, Armonk, NY).

Results: In a total of 1,296 children attending a children’s hospital in Qassim, Saudi Arabia, we found that the distribution of their age was as follows: 20.8% were one year or less, 30% were two to three years, 23.4% four to six years, and 25.8% were more than six years. The most common reasons for coming to the hospital are that 41% of children came with respiratory symptoms, 18.4% with trauma, and 15% with gastrointestinal symptoms. Respiratory infection/disease was the most commonly reported diagnosis among participants (43.5%), followed by trauma (18.6%) and gastroenteritis (15.3%). Furthermore, we found that (55.6%) refused intervention, while (39.7%) refused observation, 0.7% refused hospitalization, and 0.2% refused examination. A nasal swab was the most commonly refused procedure (28.9%), followed by blood investigations (9.9%) and IV fluids (5.7%).

Conclusion: Refusal of essential medical interventions based on evidence-based practice is still a critical issue in pediatric patients. Identifying the most frequent refused intervention and the reasons beyond intervention refusal is crucial to minimizing the refusal rates.

## Introduction

Informed consent is the permission that should be taken from the patients or their legal guardians if they are incompetent to decide on their treatment, which includes children below the age of majority, the mentally ill, and the unconscious [1]. It is obtained to ensure the patient and their surrogates receive enough information in understandable language to decide [1,2]. It is applied to conduct ethical processes and protect patients’ rights [3]. It can be done verbally asking them to sign a written consent. The patient should be competent to give valid consent, and it is made voluntarily without being influenced by others [4]. It should include the nature of the intervention, indications, benefits, possible consequences, complications, detailed alternatives, and an estimation of patient understanding of all the aspects [5].

In pediatric practice, refusal of medical intervention or treatment is possible. Treatment refusal is described as a patient's or legal guardian's outright refusal of any form of investigation, medical therapy, or surgery advised or mandated by medical specialists for a possibly curable ailment [6,7]. If the therapy is optional, there is no ethical issue with refusal. However, if the treatment is essential based on evidence-based practice, clinicians should assess the condition's seriousness and the patient's risk-benefit ratio [8]. Pressure and force should be avoided as much as possible while dealing with children and teenagers [9]. However, if the recommended action and treatment would save the kid's life or if a substantial injury will result if the patient or their guardians refuse, the physician must protect the minor [10,11]. As per Saudi health law, consent is required for medical interventions. However, in emergencies or life-threatening situations, healthcare providers may proceed without consent to save a life or prevent severe harm [12].

Communication between children, parents, and healthcare professionals is crucial for patients’ cooperation and satisfaction with medical care [13]. Involving the child in the decision of his treatment helps with compliance. Another significant aspect of participation is showing respect to the child and a sense of control. Moreover, children’s participation plays a role in their development [14].

The American Academy of Pediatrics Committee on Bioethics and the Confederation of European Specialists in Pediatrics (CESP) Ethics Working Group recommend that pediatricians provide detailed information about the diagnosis; this information should be simple, straightforward, and free of unnecessary professional jargon. They should also discuss potential risk factors and treatment side effects [9,15].

In a previous study in Turkey, hospitalization was the most common refusal of medical care, followed by invasive interventions, where lumbar puncture was the commonest refused intervention [16]. However, most studies in Saudi Arabia are limited to surveys and focus on the refusal of lumbar puncture only. In a study conducted in Taif, Saudi Arabia, parents of children who required LP refused when asked to, even when indicated [17]. Another study of children with meningitis showed parents refusing lumbar puncture [18].

This study aims to determine the frequency of refusing medical procedures and treatment in pediatric patients at a children’s hospital in Qassim.

## Materials and methods

Study design, area, population, and sampling

A retrospective review was done to evaluate and describe the informed refusal at a children's hospital in Qassim, Saudi Arabia. The data were collected from the medical records. We included all patients under 14 years and/or legal guardians who refused interventions or medical lines of treatment from January 2019 to June 2022. We excluded those incomplete files/documents, documents that lacked signed refusal forms, patients who left without signing the refusal form and were seen in OPD, and patients who were more than 14 years old. A total of 1,296 files were obtained.

Methods for data collection

We retrospectively extracted our data from patients’ medical records that fit our inclusion criteria from the hospital's database system. Then, data were collected, and to ensure data safety, the extracted data were inputted into an Excel sheet (Microsoft® Corp., Redmond, WA) and saved on a secured computer. We collected data about age, gender, information about the diagnosis, clinical notes, and treatment, which has been refused with a reason, and demographic data were not obtained as the files lacked such information.

Statistical analysis

We analyzed the collected data by the Statistical Product and Service Solutions (SPSS, version 24; IBM SPSS Statistics for Windows, Armonk, NY). Descriptive statistics were performed, and categorical variables were frequency distributions and proportions. The statistical significance between groups was tested using the chi-square test (X^2^) for categorical variables (age, gender, nationality, reason they came to ER, and diagnosis).

## Results

In a total of 1,296 children attending Qassim children's hospital in Saudi Arabia, we found that the distribution of their age was as follows: 20.8% were one year or less, 30% from 2 to < 4 years, 23.4% from four to six years, and 25.8% were more than six years. Our results demonstrated that 54.2% were males and 45.8% were females, while 98.5% of participants had Saudi nationality and 1.5% were non-Saudi (Table 1).

**Table 1:** Characteristics of the study participants (n=1,296)

Characteristic		Frequency	Percent
Age	One year or less	269	20.8%
	2 to < 4 years	389	30%
	4-6 years	303	23.4%
	More than six years	335	25.8%
Gender	Male	703	54.2%
	Female	593	45.8%
Nationality	Saudi	1,277	98.5%
	Non-Saudi	19	1.5%

In regards to the reasons of coming to ER, we found that 41% of children came with respiratory symptoms, 18.4% came with trauma, 15% came with gastrointestinal symptoms, 2.8% came with toxic ingestion, 2.1% came with allergic symptoms, and 1.8% came with CNS symptoms. In contrast, the rest of them came with other causes, such as epistaxis, decrease oral intake, and hypogylcemia (Table 2).

**Table 2:** Reasons they came to ER

Reason	Frequency	Percentage
Respiratory symptoms	531	41%
Trauma	239	18.4%
Gastrointestinal symptoms	194	15%
Toxic ingestion	36	2.8%
Allergic symptoms	27	2.1%
CNS symptoms	23	1.8%
Others	246	18.9%

Regarding final diagnosis, about 43.5% of the children were diagnosed with respiratory infections/diseases, 18.6% were diagnosed with trauma, 15.3% were diagnosed with gastrointestinal infections/disease, 2.8% had accidental poisoning, 2.4% had allergies, 2.1% were having fever, and 15.3% of children were having other problems (Table 3).

**Table 3:** Diagnosis

Diagnosis	Frequency	Percentage
Respiratory infections/disease	564	43.5%
Trauma	241	18.6%
Gastrointestinal infections/disease	198	15.3%
Accidental poisoning	36	2.8%
Allergy	31	2.4%
Fever	27	2.1%
Others	199	15.3%

In regards to the refused procedures and interventions in general, we found that 55.5% refused intervention, 39.7% refused observation, 0.7% refused hospitalization, 0.2% refused examination, and 3.9% refused other things concerning about type of procedure.

**Figure 1:**
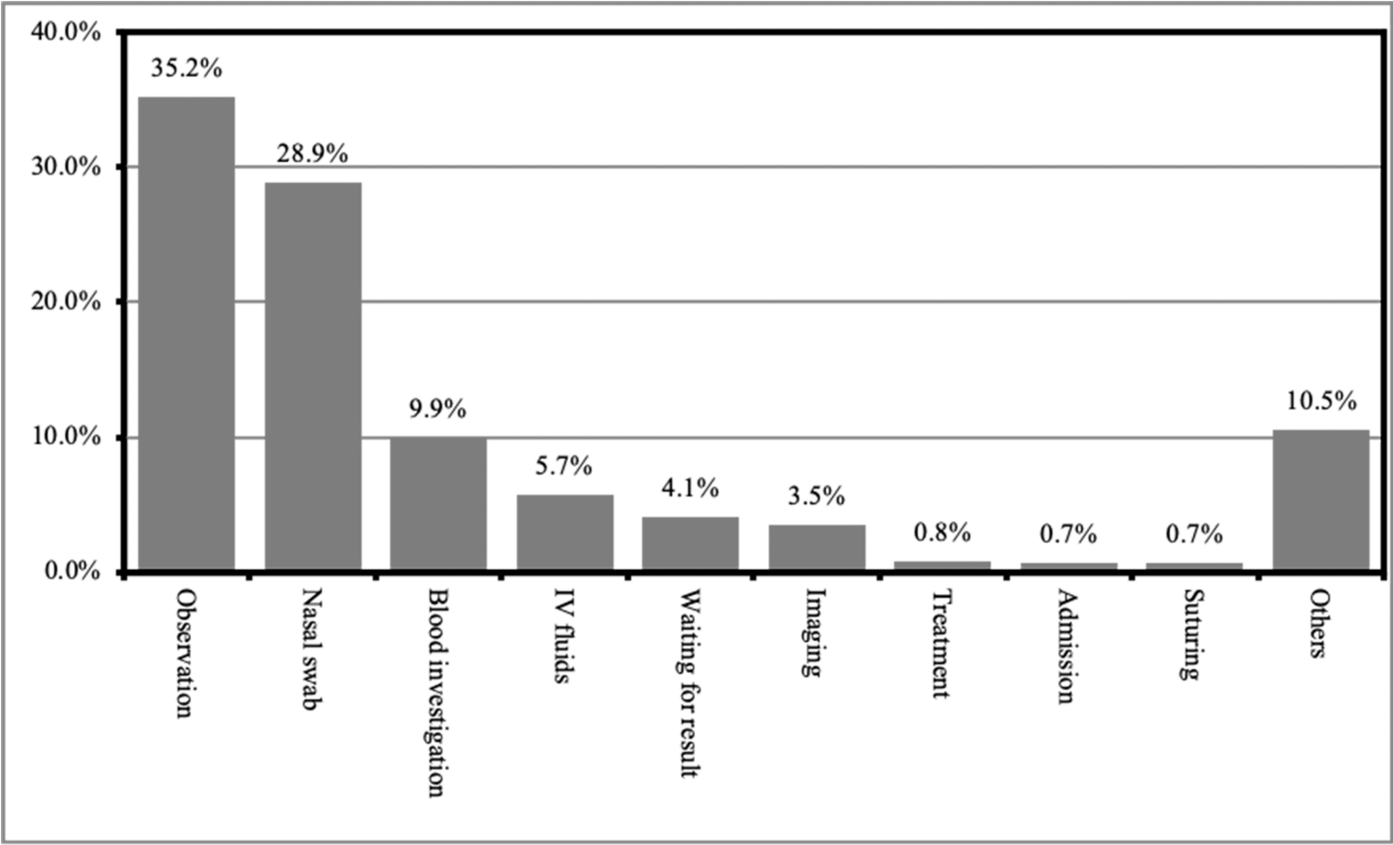
Procedures refused by the participants

More specifically, interventions refused by the participants are as follows: 35.2% refused observation, 28.9% refused nasal swab check for respiratory infection, 9.9% refused blood investigations, 5.7% refused IV fluids, 4.1% refused even waiting for results, 3.5% refused imaging, 0.8% refused treatment, 0.7% refused admission and suturing, and more than 10% of participants refused other procedures (Figure 1).

## Discussion

The current study aimed to assess the frequency of refusal of medical procedures and treatment in pediatric patients at a children's hospital in Qassim, Saudi Arabia. In pediatric practice, patients may refuse medical intervention or treatment.

Treatment or procedure refusal is defined as the patient's or legal guardian's outright refusal of any investigation, medical care, or surgery recommended or ordered by medical professionals for a potentially curable disease [6,7]. This study was the first to assess the refusal of medical care in pediatric practice in Saudi Arabia.

In this current study, we found that the refusal of medical management was frequent in pediatric practice. The most commonly reported reason for refusal of medical management was interventions, followed by observation. This is inconsistent with another study conducted in Turkey, which showed that the most common refused recommendation was hospitalization, which was the least reported reason in our study [19].

A previous study in China showed different reasons for the refusal of medical management, such as financial difficulty, transportation difficulties, and fear of severe side effects [20]. These variations are most likely due to differences in the healthcare system as medical care in Saudi Arabia is governmental and free to Saudis, as well as due to the population's economic status between countries.

The results of this study found that nasal swab was the most commonly refused procedure. In contrast, a previous study revealed that lumbar puncture was the most frequently refused procedure [16]. This is most likely due to differences in the medical conditions of the different sampled populations.

Additionally, these findings demonstrated that respiratory tract infection was the most commonly reported diagnosis among participants. However, an earlier study revealed neonatal sepsis was the most frequent diagnosis [21]. This is clearly due to significant variations in the age of the sampled population between the two studies.

This research had some limitations. The data were extracted from electronic medical records, which rely on administrative and medical staff accurately entering data. Furthermore, this study lacked patient follow-up data, focusing on descriptive data and a correlation study between relevant factors. This finding provides a need for further studies to explore the reason for refusal from the patient and family perspective.

We recommend that more research be conducted with a more diverse and representative population and systemic health education for parents and communities. Effective communication between physicians and parents is required.

## Conclusions

Refusal of essential medical interventions based on evidence-based practice is still a critical issue in pediatric patients. Identifying the most frequent refused intervention and the reasons beyond intervention refusal is crucial to minimizing the refusal rates. This study showed that refusing medical care is a common problem and that intervention followed by observation was the most common.
